# Development, Problem Behavior, and Quality of Life in a Population Based Sample of Eight-Year-Old Children with Down Syndrome

**DOI:** 10.1371/journal.pone.0021879

**Published:** 2011-07-21

**Authors:** Helma B. M. van Gameren-Oosterom, Minne Fekkes, Simone E. Buitendijk, Ashna D. Mohangoo, Jeanet Bruil, Jacobus P. Van Wouwe

**Affiliations:** Department Child Health, Netherlands Organization for Applied Scientific Research (TNO), Leiden, The Netherlands; The University of Queensland, Australia

## Abstract

**Objective:**

Children with Down syndrome (DS) have delayed psychomotor development. We investigated levels of development, problem behavior, and Health-Related Quality of Life (HRQoL) in a population sample of Dutch eight-year-old children with DS. Developmental outcomes were compared with normative data of eight-year-old children from the general population.

**Method:**

Over a three-year-period all parents with an eight-year-old child with DS were approached by the national parent organization. Developmental skills were assessed by means of the McCarthy Scales of Children's Ability. To measure emotional and behavioral problems we used the Child Behavior Checklist. HRQoL was assessed with the TNO-AZL Children's Quality of Life questionnaire. Analyses of variance were applied to compare groups.

**Results:**

A total of 337 children participated. Mean developmental age was substantially lower than mean calendar age (3.9 years, SD 0.87 and 8.1 years, SD 0.15 respectively). Mean developmental age was significantly lower among boys than girls (3.6 (SD 0.85) and 4.2 years (SD 0.82) respectively; p<0.001). Compared with the general population, children with DS had more emotional and behavioral problems (p<0.001). However on the anxious/depressed scale, they scored significantly more favorably (p<0.001). Significantly lower HRQoL scores for the scales gross motor skills, autonomy, social functioning and cognitive functioning were found (p-values<0.001). Hardly any differences were observed for the scales physical complaints, positive and negative emotions.

**Conclusion:**

Eight-year-old children with DS have an average developmental delay of four years, more often have emotional and behavioral problems, and have a less favorable HRQoL compared with children from the general population.

## Introduction

An important feature of children with Down syndrome (DS) is their delayed development. DS is known as the most prevalent cause of intellectual impairment associated with a chromosomal anomaly (Trisomy 21). In the United States prevalence of DS is estimated to be 12 per 10,000 live births; in 2002 83,400 children with DS aged 0–19 years lived in the United States [1]. In the Netherlands the prevalence of DS seems higher: 14 per 10.000 live births (approximately 270 children per year) [2]–[4]. Children with DS have a well-recognized phenotype, including external characteristics, specific physical problems (such as congenital heart defects, gastro-intestinal disorders, thyroid dysfunction and visual impairment) and intellectual impairment with delayed cognitive and motor development [5], [6].

This delayed development has frequently been studied during the past 50 years. Results indicated that children with DS have a lower IQ and have difficulties with expressive language [7]–[14]. In particular, they have difficulties with verbal working memory, receptive language, reading, writing and arithmetic [7], [8], [12], [14], [15]. Studies on behavior problems indicate on average one quarter to one third of the children with DS to have significant emotional and behavior problems [6], [10], [16]. Most studies observe that children with DS frequently have speech problems, attention deficit and concentration problems, social withdrawal, stubbornness, as well as oppositional and disobedient behavior [9]–[11], [16], [17]. A substantial number of studies found that 8–23% of the children with DS have significant psychopathology [10]. Specifically, 7% are diagnosed with autism, 6% to 9% with attention deficit/hyperactivity disorder (ADHD), and 10% to 15% with conduct or oppositional disorders [6], [10], [16]–[20].

The vast majority of the above-mentioned studies are not population based and included fewer than 50 children with DS. Studies that included larger numbers of children with DS date back to the 1970s and 1980s, when the children grew up under different circumstances. Improvement of medical care and general support could have enhanced overall development of children with DS or specific aspects of their development. Our sample is studied between 2000 and 2003, and since no major changes in the approach to the developmental aid, medical care and upbringing of children with DS have taken place since then, in our opinion these results are still valid.

When a child with DS is born, parents want to be informed reliably on the expected development of their child. Most of the current available information focuses on the medical aspects like concomitant congenital anomalies and organic disorders, which children with DS are at high risk for. Hardly any information is available on the actual expected development. In this study we aimed to investigate the developmental skills, problem behavior, and Health-Related Quality of Life (HRQoL) in a large population based sample of Dutch children with DS at the age of eight years old, compared to normative data from same-age-children from the general population. Subsequently, we aimed to provide valuable information for families, health care and educational professionals all involved in the care for children with DS.

## Methods

### Ethics statement

The study protocol was approved by the Medical Ethics Committee of the Leiden University Medical Centre, and written informed consent was obtained from parents/next of kin of all participants.

### Subjects

Dutch families, who were member of the Dutch Down Syndrome Foundation and who parent a child with DS born in 1992, 1993 or 1994, were invited to participate in the study. The Dutch Down Syndrome Foundation manages a database which includes most Dutch children with DS. In the Netherlands, about 80% of all parents with a child with DS in the age up to 12 year join this organization, routinely advised by their pediatrician [21].

The selected parents received a written request from the Dutch Down Syndrome Foundation to participate. Parents, who signed up to participate, received a set of questionnaires and an appointment for a home visit. Between June 2000 and February 2003 a professional, experienced and trained psychological assistant visited the children at home and administered the McCarthy Scales of Children's Ability (MSCA) [22]. Developmental testing was conducted following their eighth birthday. If the test was not completed during one visit, the child was rescheduled for further testing within a few weeks. The set of parent questionnaires contained two formal tests: the Child Behavior Checklist (CBCL) and the TNO-AZL Children's Quality of Life (TACQOL) [23]–[25]. Questions on background, demographic variables and medical condition of the child were included in the set of questionnaires.

### Measures

The Dutch version of the MSCA developed for children aged 2.5 to 8.5 years was used [26]. The MSCA contains 18 subtests, grouped into the scales: verbal, perceptual, quantitative, memory and motor skills. The verbal, perceptual and quantitative scales are combined to form the general cognitive scale. A developmental age is calculated based on the various scale scores. To prevent an excessive influence of one of the subscales on the developmental age, the 18 subtests are each representing one competence in order to test a specific ability of the child and not a broad range of abilities, i.e. the test is developed so that level of verbal ability will minimally influence test-scores on other domains measured.

The CBCL measures emotional and behavior problems. The Dutch version of the CBCL for four to twelve years old children was used [27]. The CBCL is suitable for children with developmental delay [28]–[30]. It contains 118 problem behavior items rated from 0 (not true) to 2 (very true or often true), covering nine scales: withdrawn, somatic complaints, anxious/depressed, social problems, thought problems, attention problems, delinquent behavior, aggressive behavior and sexual problems. These scales combined form the scales internalizing problems (containing withdrawn, somatic complaints and anxious/depressed) and externalizing problems (containing delinquent and aggressive behavior).

The TACQOL, developed in the Netherlands for children aged six to fifteen years old, was used to measure HRQoL. It assesses functional problems weighted by the degree to which a child shows negative emotions to such problems. The questionnaire contains 56 items divided over seven scales: physical complaints, gross motor skills, autonomy, cognitive functioning, social functioning, positive emotions and negative emotions.

### Statistical analysis

All questionnaires and test results were collected and merged into one dataset. The levels of developmental skills, problem behaviour and HRQoL were calculated for each outcome measure, according to the accompanying (supplementary) test manuals. The test results of the DS-sample were – for each outcome measure – compared with normative data from the general population, matched on calendar age. Analyses of variance (ANOVA) were used to evaluate differences between mean values. All statistical tests were 2-tailed and statistical significance was defined at *p*<0.05. The effect sizes were estimated by dividing the differences in mean scores between the subgroups by the pooled SD. Cohen's effect sizes (*d*) were used for interpretation of relevant differences: *d*<0.2 is considered a negligible difference, 0.2≤*d*<0.5 a small, 0.5≤*d*<0.8 a moderate, 0.8≤*d*<1.3 a large, and *d*≥1.3 a very large difference [31]. Means for the total DS-sample were weighted for gender. All analyses were performed using Statistical Package for the Social Sciences, version 17.0 for Windows (SPSS Inc, Chicago, Illinois).

Analyses of the MSCA scales were carried out with raw scale scores. The norms for children of 8.25 years of age, as available in the accompanying manual and based on a nationwide sample from the United States, were used for comparison with the DS-sample [22]. In addition, developmental age was calculated and compared for boys and girls, using ANOVA.

For the CBCL, normative data were derived from a Dutch sample of 661 children aged seven to nine years (mean 7.9 years, SD 0.40) [32]. In addition to the comparison of scale scores, the proportions of subjects with a scale score in the clinical area were compared between DS-sample and the normative sample, using Chi-Square test.

The TACQOL was analyzed by comparing the scale scores of the DS-sample with data from a Dutch reference population of 519 children aged eight or nine years [25].

## Results

A total of 531 parents with a child with DS born in 1992, 1993 or 1994 were approached via the Dutch Down Syndrome Foundation to participate in the present study, which holds 78% of all living children of this birth cohort in the Netherlands (based on a 84% survival rate) [33]. A total of 380 parents (72%) signed up to participate; 337 parents provided actual data for the present study (response rate: 63%; equaling 50% of this birth cohort). Gender and age were known of all 337 participating children. Background characteristics were known of 325 children. The number of subjects participating in the formal tests was 325 for the TACQOL, 320 for the CBCL and 285 for the MSCA. Overall, 266 parents and their children completed all questionnaires and tests.

Background characteristics are presented in Table 1. Mean age was 8.1 years (SD = 0.15, range 7.8–9.1); 52.0% of the subjects were boys and 94.6% of the children were of Dutch origin. A total of 156 children (48.0%) attended regular education at the age of eight years; more girls than boys (60.3% versus 36.7%; *p*<.001). Above 90% of the children with DS were diagnosed and/or treated for one or more concomitant chronic diseases, mainly visual impairment, chronic airway infection, heart defect or hearing impairment.

**Table 1:** Characteristics of the studied population of children with DS, as reported by their parents, arranged by gender (n = 325).

	Total		Boys		Girls		
General characteristics	n	*%*	n	*%*	n	*%*	*p* *
Number of subjects	325	*100.0*	169	*52.0*	156	*48.0*	
Education at 8 years old							
Regular primary school	156	*48.0*	62	*36.7*	94	*60.3*	.000
Special school or day-care centre	169	*52.0*	107	*63.3*	62	*39.7*	.000
Ever enrolled in regular primary school	240	*73.8*	112	*66.3*	128	*82.1*	.001
Level of regular primary school at 8 years old							
Preschool	31	*9.5*	17	*10.1*	14	*9.0*	.614
First grade	95	*29.2*	35	*20.7*	60	*38.5*	.000
Second grade	30	*9.2*	10	*5.9*	20	*12.8*	.032
Age in years (range)	7.8 – 9.1		7.9 – 9.1		7.8 – 9.0		
Age in years (mean ± SD)	8.14 ± 0.15		8.15 ± 0.15		8.13 ± 0.15		.193
Concomitant chronic diseases							
Visual impairment	158	*48.6*	76	*45.0*	82	*52.6*	.172
Chronic Airway Infections	149	*45.8*	85	*50.3*	64	*41.0*	.094
Congenital heart defect	137	*42.2*	63	*37.3*	74	*47.4*	.064
Hearing impairment	98	*30.2*	55	*32.5*	43	*27.6*	.330
Gastrointestinal tract abnormality	45	*13.8*	27	*16.0*	18	*11.5*	.248
Thyroid dysfunction	39	*12.0*	22	*13.0*	17	*10.9*	.558
Asthma	34	*10.5*	25	*14.8*	9	*5.8*	.008
Diabetic Mellitus	3	*0.9*	2	*1.2*	1	*0.6*	.611
Other chronic disease	81	*24.9*	46	*27.2*	35	*22.4*	.758
Number of concomitant chronic diseases							
No chronic disease	26	*8.0*	18	*10.7*	8	*5.1*	.067
Only one chronic disease	81	*24.9*	39	*23.1*	42	*26.9*	.423
Two or more chronic diseases	218	*67.1*	112	*66.3*	106	*67.9*	.748

Abbreviation: SD – standard deviation.

* Boys with DS compared to girls with DS.

### Developmental skills

The psychological assistants visited 285 children at home to administer the MSCA. Table 2 shows mean scores of the test scales. Higher values reflect more favorable results.

**Table 2:** Developmental skills, measured by the MSCA in a population of eight-year-old children with DS (n = 285), compared to the normative sample (NS, n = 238); raw scale scores are reported; higher scores denote more favorable skills.

	Total sample (male and female)			DS sample		
	DS	NS		Male	Female	
	*(n = 285)*	*(n = 106)*		*(n = 153)*	*(n = 132)*	
	Mean ± SD	Mean ± SD	Effect size^1^	Mean ± SD	Mean ± SD	Effect size^1^
Verbal	37.32±18.11	89.00±10.75	−3.15***	33.29±18.73	41.36±16.57	−0.46***
Perceptual-Performance	29.84±15.89	76.00±7.00	−3.29***	24.88±15.69	34.80±14.53	−0.66***
Quantitative	10.98±6.78	44.00±7.00	−4.84***	9.09±6.71	12.86±6.33	−0.58***
Memory	12.56±7.81	50.00±6.25	−5.06***	10.52±7.30	14.60±7.80	−0.54***
Motor	25.79±21.42	64.00±5.50	−2.07***	22.35±12.49	29.23±11.39	−0.58***
General Cognitive	78.15±38.13	209.00±20.00	−3.84***	67.26±38.37	89.05±34.75	−0.60***

* p<0.05,

** p<0.01,

*** p<0.001.

1 Cohen's *d* effect size: *d*<0.2 negligible; 0.2≤*d*<0.5 small; 0.5≤*d*<0.8 moderate; 0.8≤*d*<1.3 large; *d*≥1.3 very large.

Abbreviations: MSCA – McCarthy Scales of Children's Ability, DS – Down syndrome, NS – Normative sample, SD – standard deviation.

On all measures the DS-sample scored significantly lower than the normative sample, with very large effect sizes. The largest effect sizes were found for the memory scale (*d* = 5.1) and the quantitative scale (*d* = 4.8). Within the DS-sample, boys had significantly lower scale scores than girls; effect sizes ranged from 0.5 to 0.7, indicating a moderate effect by gender.

**Figure 1:**
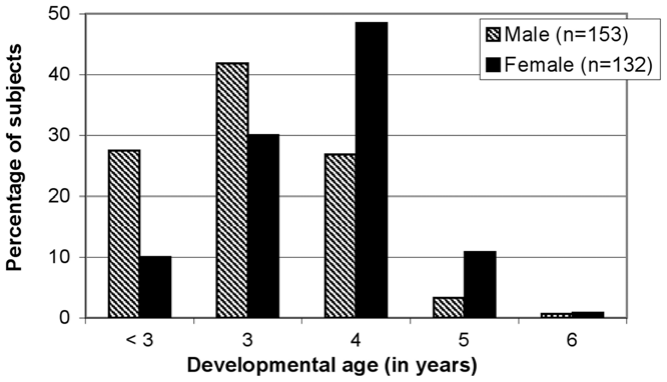
Distribution of MSCA developmental age in eight-year-old children with DS, arranged by gender (n = 285).

In addition, developmental age was calculated for each child. In Figure 1 the developmental age of the subjects is plotted stratified for gender. In 33 children (82% boys), who had very low test scores, developmental age could not be calculated exactly, but was estimated to be at a level of under 2.5 years, according to the MSCA test manual. By calculating mean age these children were ranked as having a developmental age of 2.4 years. For the total sample, mean developmental age for boys was 3.6 years (SD = 0.85) and for girls 4.2 years (SD = 0.82), showing a difference of 0.53 years (*p*<0.001). In all children the highest developmental age scored was 6.8 years.

### Emotional and behavior problems

A total of 320 questionnaires of the CBCL were completed. Mean scores are shown in Table 3, whereby higher values reflect more problems. On almost all subscales children in the DS-sample scored significantly more problem behavior than the normative sample. The highest effect size was found for the subscale social problems (*d* = 1.55), followed by the subscale attention problems (*d* = 1.15 for boys and 1.30 for girls), indicating large to very large effects. The effect size of the difference in total problem score between the children with DS and the normative sample indicated a moderate effect (*d* = 0.72 for boys and *d* = 0.63 for girls).

**Table 3:** Emotional and behavioral problems, measured by the CBCL, in a population of eight-year-old children with DS (n = 320): scale scores and proportion of children scoring in the clinical area of the scales, compared to the normative sample (NS, n = 661), arranged by gender; higher scores denote more problems.

	Total DS sample		Male					Female				
	Scale score	Clinical score	Scale score			Clinical score		Scale score			Clinical score	
	*(n = 320)*		DS *(n = 169)*	NS *(n = 325)*		DS	NS	DS *(n = 151)*	NS *(n = 336)*		DS	NS
	Mean ± SD	%	Mean ± SD	Mean ± SD	Effect size^1^	%	%	Mean ± SD	Mean ± SD	Effect size^1^	%	%
Withdrawn	2.54±2.66	5.9	2.61±2.75	1.63±1.85	0.45***	7.1**	1.5	2.46±2.55	1.65±1.84	0.39***	4.6	2.1
Somatic complaints	1.35±1.92	6.9	1.29±1.85	0.77±1.24	0.35***	7.7**	2.2	1.42±2.00	1.04±1.49	0.23*	6.0*	2.1
Anxious/Depressed	0.88±1.42	0.3	0.78±1.33	2.40±3.20	−0.60***	0.6*	4.3	0.99±1.50	2.50±3.26	−0.53***	0.0*	3.6
Social problems	4.38±2.15	21.6	4.57±2.17	1.51±1.83	1.57***	18.9***	1.9	4.17±2.11	1.30±1.76	1.53***	24.5***	2.7
Thought problems	1.18±1.66	8.8	1.30±1.76	0.35±0.92	0.75***	10.7***	1.9	1.03±1.53	0.27±0.67	0.75***	6.6***	0.9
Attention problems	6.50±3.15	12.2	6.76±3.19	3.22±3.02	1.15***	12.4***	2.5	6.22±3.10	2.61±2.62	1.30***	11.9***	2.1
Delinquent behavior	1.47±1.57	3.1	1.55±1.60	1.11±1.43	0.30**	2.4	1.5	1.38±1.53	0.78±1.23	0.45***	4.0	1.5
Aggressive behavior	7.27±5.69	4.4	8.13±6.06	6.66±6.11	0.24*	5.9	4.3	6.30±5.11	5.07±5.05	0.24*	2.6	2.4
Sexual problems	0.37±0.83	3.8	0.42±0.89	0.15±0.52	0.40***	3.6	1.2	0.30±0.76	0.11±0.42	0.35***	4.0**	0.3
Internalizing problems^2^	4.71±4.55	10.6	4.62±4.54	4.69±4.96	-	11.2	11.4	4.82±4.58	5.09±5.32	-	9.9	11.0
Externalizing problems^3^	8.73±6.84	15.3	9.68±7.25	7.77±7.17	0.27**	12.4	11.1	7.68±6.21	5.85±5.95	0.30**	18.5	13.4
Total problems	30.08±18.06	26.9	32.05±18.81	19.78±15.96	0.72***	27.8***	10.8	27.87±16.97	17.98±15.28	0.63***	25.8***	11.9

*p<0.05,

**p<0.01,

***p<0.001.

1 Cohen's *d* effect size: *d*<0.2 negligible; 0.2≤*d*<0.5 small; 0.5≤*d*<0.8 moderate; 0.8≤*d*<1.3 large; *d*≥1.3 very large.

2 Combined from the subscales withdrawn, somatic complaints and anxious/depressed.

3 Combined from the subscales delinquent and aggressive behavior.

Abbreviations: CBCL – Child Behavior Checklist, DS – Down syndrome, NS – Normative sample, SD – standard deviation.

For the scale anxious/depressed an opposite score was observed: the DS-sample showed significantly fewer problems with a moderate effect size (*d* = 0.60 for boys and *d* = 0.53 for girls).

Additionally, scale scores were arranged in the normal or clinical area of the scale (Table 3). For the subscales somatic complaints, social, thought, attention and sexual problems, and the total problem scale the children with DS scored significantly more within the clinical areas. An exception again was the subscale anxious/depressed, where fewer children with DS scored within the clinical area (*p*<0.05).

### Health-Related Quality of Life

Mean scores of the TACQOL – evaluating the influence of having DS in daily life – are summarized in Table 4, whereby higher values reflect better HRQoL. Compared with the normative population, children with DS had significantly lower scores with very large effects on autonomy (*d* = 1.90) and cognitive functioning (*d* = 1.51); with a large effect on the gross motor skills (*d* = 0.89), and with a small effect on social functioning (*d* = 0.46) scales.

**Table 4:** HRQoL, measured by the TACQOL, in a population of eight-year-old children with DS (n = 325), compared to the normative sample (NS, n = 519), arranged by gender; higher scores denote better HRQoL.

	Total DS sample		Male			Female	
		DS	NS		DS	NS	
	*(n = 325)*	*(n = 169)*	*(n = 260)*		*(n = 156)*	*(n = 259)*	
	Mean ± SD	Mean ± SD	Mean ± SD	Effect size^1^	Mean ± SD	Mean ± SD	Effect size^1^
Physical complaints	27.26±3.48	27.14±3.56	27.29±3.92	-	27.39±3.39	26.66±4.05	-
Gross motor skills	27.85±3.92	27.76±4.21	30.78±2.50	−0.90***	27.95±3.61	30.72±2.85	−0.88***
Autonomy	26.28±3.59	26.06±3.72	31.10±1.90	−1.84***	26.51±3.45	31.32±1.60	−1.96***
Cognitive functioning	22.76±3.54	22.75±3.89	28.39±3.98	−1.43***	22.77±3.12	28.62±4.00	−1.58***
Social functioning	28.25±3.54	27.95±3.72	29.36±2.82	−0.44***	28.57±3.33	29.91±2.49	−0.47***
Positive emotions	15.06±1.64	15.01±1.70	14.73±2.01	-	15.11±1.58	14.81±2.05	-
Negative emotions	11.71±1.99	11.70±2.18	11.18±2.78	0.21	11.73±1.78	11.45±2.30	-

* p<0.05,

** p<0.01,

*** p<0.001.

1 Cohen's *d* effect size: *d*<0.2 negligible; 0.2≤*d*<0.5 small; 0.5≤*d*<0.8 moderate; 0.8≤*d*<1.3 large; *d*≥1.3 very large.

Abbreviations: HRQoL – Health-related quality of life, TACQOL – TNO-AZL Children's Quality of Life questionnaire, DS – Down syndrome, NS – Normative sample, SD – standard deviation.

For the scale negative emotions we observed a significant favorable outcome for boys, but the effect size was small (*d = 0.21*). For the other scales – physical complaints, positive emotions and (for girls) negative emotions – there were no differences observed.

## Discussion

The aim of the present study was to investigate the development of a population based sample of Dutch children with DS at the age of eight years. The study included a large nationwide cohort of children with DS born in 1992, 1993 or 1994, approximately 50% of all children with DS of this birth cohort living in the Netherlands [2]–[4]. We measured a wide spectrum of developmental skills, emotional and behavior problems and HRQoL. Previous studies indicated that children with DS have a delayed development [7]–[12], [14], [15]. However, detailed aspects of development and quality of life have not been quantified in a population based sample.

The large sample of our study presented an opportunity to study differences between boys and girls. Few studies addressed gender differences with regard to developmental skills and cognition. Most observed no significant differences, probably due to their small sample size [10].

Furthermore the present study is different from most others in defining the comparison groups. Previous studies compared their DS-sample with participants matched for mental age and gender or used siblings [9]–[12], [17]. We compared the selected DS-sample with randomly selected children from the general population with identical chronological age and same gender.

Our sample is born in 1992, 1993 and 1994, after the era (the 1980's) in which major changes in the care for the children with DS have taken place; i.e. medical care has been optimized; early intervention was introduced, and the majority of the children was no longer raised in institutions but at home.

### Results

One of the main findings of our study is that children with DS have a substantial delay in developmental skills in comparison with the normative sample. The mean developmental age of the children with DS was 3.9 years (SD = 0.87), which is four years behind their average calendar age of 8.1 years (SD = 0.15). A substantial delay in development was recorded in all children. However, the range was wide, with some children indicating a developmental delay of only one or two years, and other children indicating a developmental delay of more then five years. Girls with DS scored on average more favorably on all skills and had a higher developmental age in comparison to boys with DS.

Our results further indicate that children with DS had more emotional and behavior problems in comparison to the normative sample on almost all domains measured, with the exception of the scale anxious/depressed. Some previous studies with a small number of children (less than 50) and a wide range of age, found also more behavior problems in children with DS [11]. Our finding that children with DS score significantly better on the problem scale anxious/depressed in comparison to the normative sample has not been reported in previous studies [9], [11], [16]. Only among adults with DS, several studies report more depression compared to the general population [6], [18], [19]. The internalizing problem scale score – which is composed of withdrawn, somatic complaints and anxious/depressed – did not differ significantly from the normative sample, as a result from the opposite score for the anxious/depressed scale.

The children with DS scored a lower HRQoL on the scales gross motor skills, autonomy, social and cognitive functioning in comparison with the normative sample. These domains determine the main topics in everyday life of children with DS, as reported by parents. Remarkably, HRQoL of children with DS showed no significant difference on the physical complaints scale, even though 92% of the children indicated one or more concomitant chronic conditions.

In our sample a high percentage (46%) of chronic airway infections was observed. Prospective studies on the exact incidence of chronic airway infections are lacking. A recent national health survey showed that in children with DS in the age of 6–10 years parents reported 38% to have head or chest cold in the previous two weeks [34]. A study among Dutch children showed that 24% of the children with DS were hospitalized twice or more for pulmonary infection or use inhalation medication for more than six months [35]. In our study parents were asked “Does your child suffer from chronic airway infections (often severe common cold/bronchitis)” and “Was your child diagnosed with asthma?” to evaluate respiratory complaints. Therefore, the higher number of reported chronic airway infections in our study could be explained by the broader definition used. For other concomitant disorders, like congenital heart defects, hearing impairment and thyroid dysfunction, the incidences in our sample are in accordance with earlier studies [6].

The data of this study were collected between 2000 and 2003. Despite this data collection occurred several years ago, these data will be valid for the current generation of children with DS, because no major changes in the approach of (medical) care for children with DS has taken place since the measurement of this study.

Of the parents with a child with DS who were approached, about 63% participated in the study. Unfortunately, we were not able to carry out a non-response analysis. It is possible that parents whose child had more serious developmental problems than most of the children with DS, more frequently refused to participate. These factors may have resulted in an underestimation of the problem behavior of these children and an overestimation of the developmental level. However, we observed a wide range in developmental level and more than 50 children to have a developmental age below three years, showing also children with more serious developmental problems are included in our study.

### Implications

The results of this study provide reference information for pediatricians and other health care professionals when they inform parents of the expected development of a child with DS. They may assist parents in gaining realistic expectations about the future of their children.

Currently children with DS in the Netherlands are encouraged to attend regular education in primary school. Studies showed that attending regular education provides more positive peer relationships and can improve social skills [36], [37]. However, low mean developmental age at the calendar age of eight years can be an important obstacle to enroll them in regular primary education. In order to keep them in regular education, additional support is needed to provide adequate learning conditions. If adequate support can not be guaranteed, special education is needed.

The children with DS indicated more social, attention and thought problems than the normative sample. These problems should be recognized as they form obstacles in learning conditions. In particular the high level of social problems suggests that this is an area where significant improvement may be made. From an early age onwards, children with DS should be stimulated to develop social skills, confirming the need for adequate support in primary school.

For the children themselves HRQoL was mainly decreased for their level of autonomy and cognitive functioning. Current medical care for children with DS focuses on the physical conditions of the children, as advised in the guidelines of the American Academy of Pediatrics, and the Pediatric Association of the Netherlands [38], [39]. Medical professionals should extend their care with supportive coaching on autonomy and cognitive functioning, to improve quality of life for the children with DS.

### Conclusion

Eight-year-old children with DS have an average developmental delay of four years. This finding has important implications for (parents of) children with DS and professionals. These children have more emotional and behavioral problems, and have on some domains a less favorable HRQoL compared with children from the general population.

It is recommended to investigate the factors influencing the social participation and development of children with DS, as well as the relation between developmental level and problem behavior, and its influence on quality of life. Population based longitudinal cohort studies are needed to gain insights in all aspects of functioning and social participation of children with DS.
